# Supplementary material for: Al Foil-Supported Carbon Nanosheets as Self-Supporting Electrodes for High Areal Capacitance Supercapacitors
Source: Molecules. 2023 Feb 15;28(4):1831. doi: 10.3390/molecules28041831 (PMC9966967; doi:10.3390/molecules28041831)
Supplement: Supplementary file 1 [file molecules-28-01831-s001.zip › molecules-2211778-supplementary.pdf]

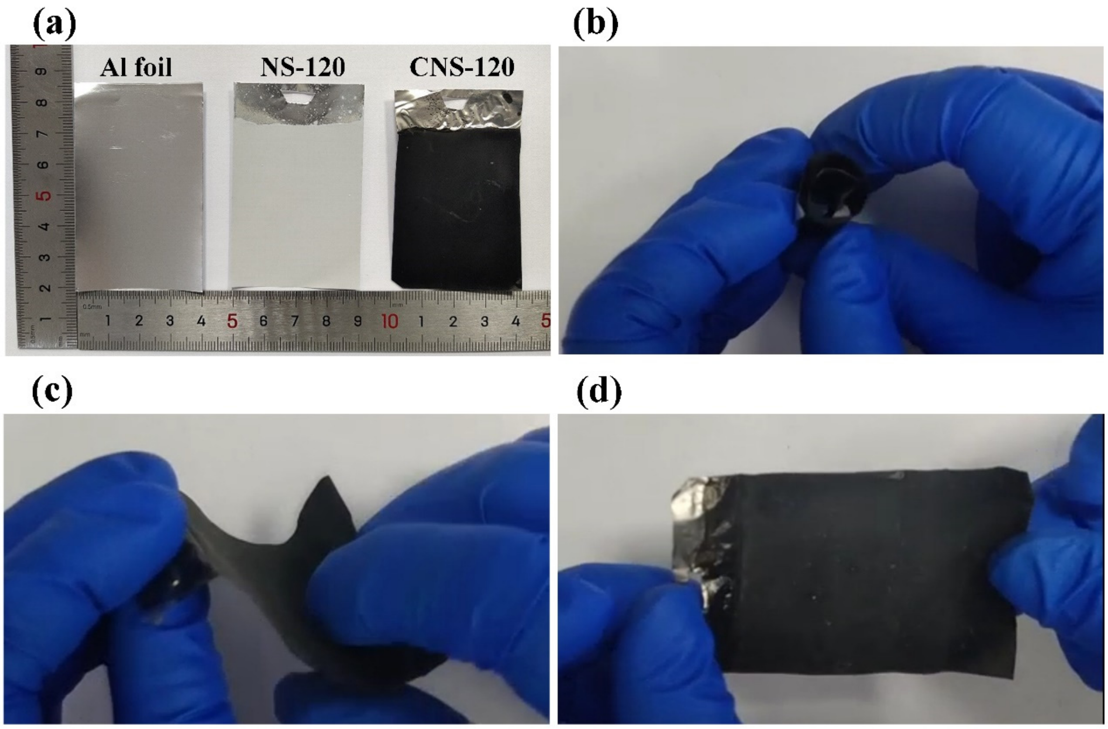

Figure S1. (**a**) digital photos of Al foil, NS-120 and CNS-120. (**b-d**) digital photos of CNS-120.

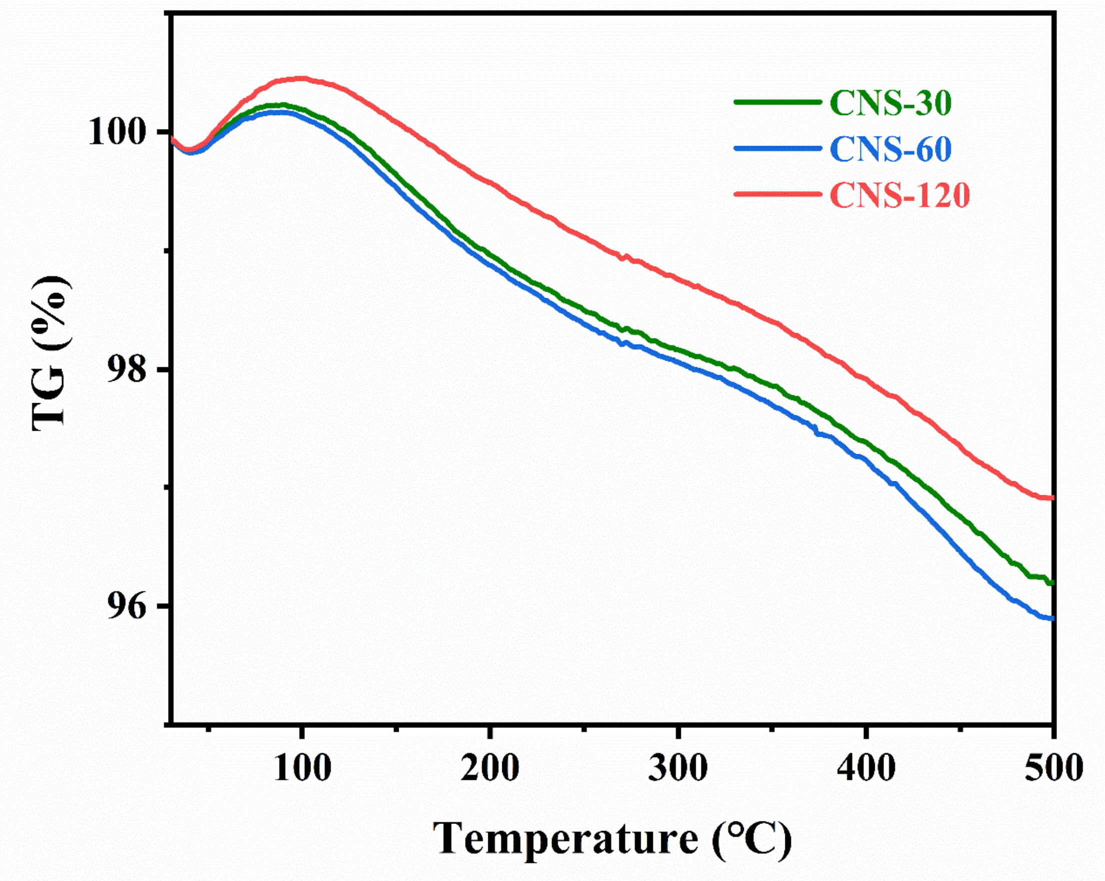

Figure S2. The TGA curves of CNS-30, CNS-60 and CNS-120.

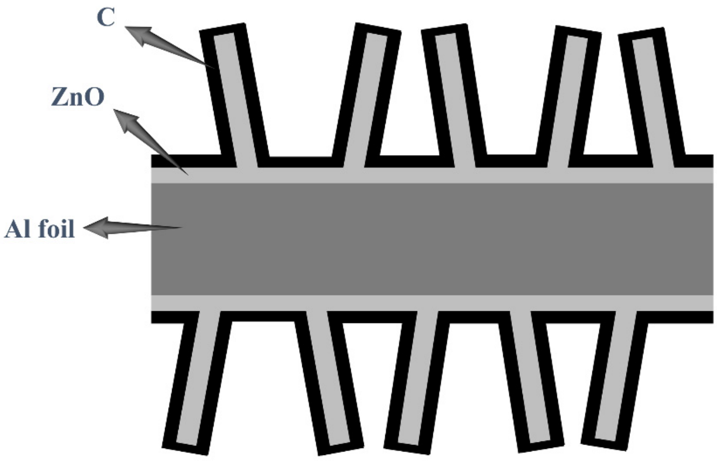

Figure S3. Schematic diagram describes the microstructure of the Al/ZnO/C self-supporting electrode.

Table S1. Element content of CNS-120.

Sample	C (at.%)	N (at.%)	O (at.%)
CNS-120	3.7	0.21	2.56

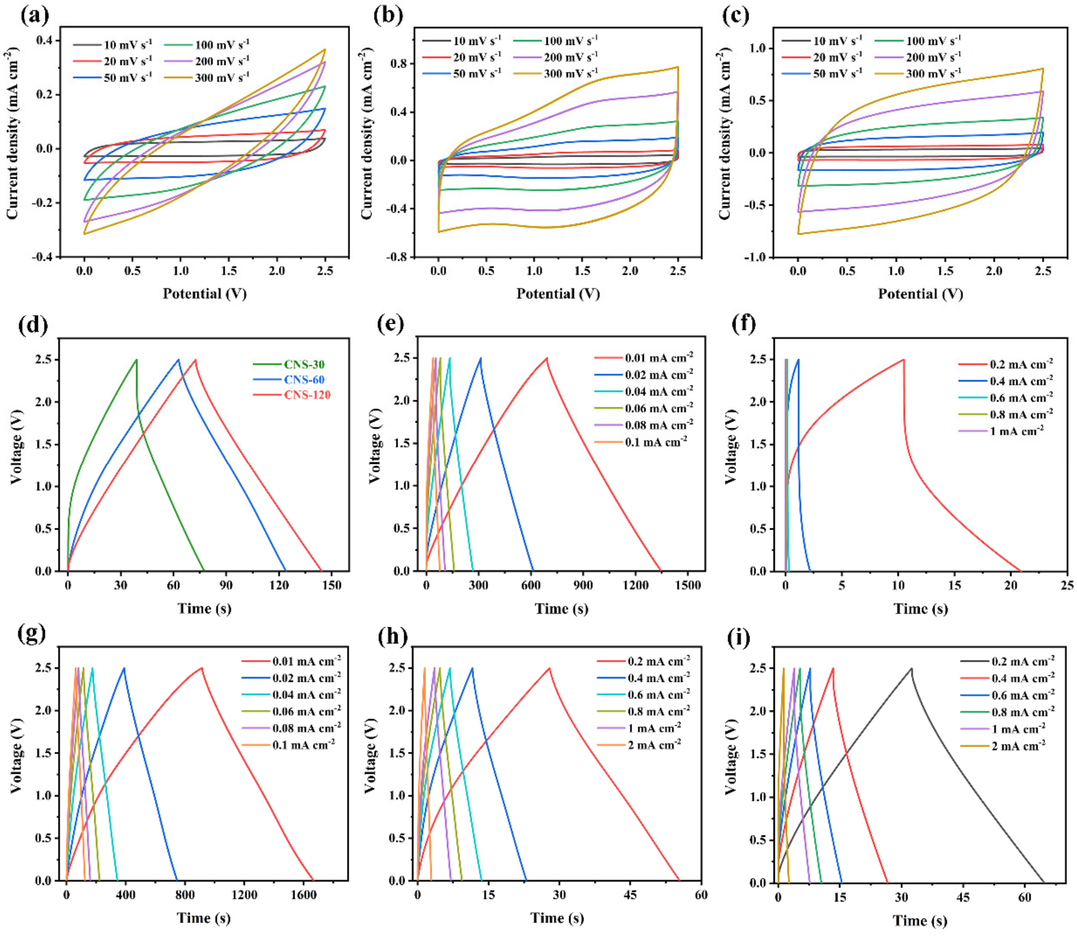

Figure S4. Electrochemical performance of CNS-30, CNS-60 and CNS-120. (a-c) CV curves of CNS-30, CNS-60 and CNS-120 at the scan rates from 10 to 300 mV s^{-1}. (d) GCD curves of CNS-30, CNS-60 and CNS-120 at the current density of 0.1 mA cm^{-2}. (e, f) GCD curves of CNS-30 at the current densities from 0.01 mA cm^{-2} to 1 mA cm^{-2}. (g, h) GCD curves of CNS-60 at the current densities from 0.01 mA cm^{-2} to 2 mA cm^{-2}. (i) GCD curves of CNS-120 at the current densities from 0.2 mA cm^{-2} to 2 mA cm^{-2}.

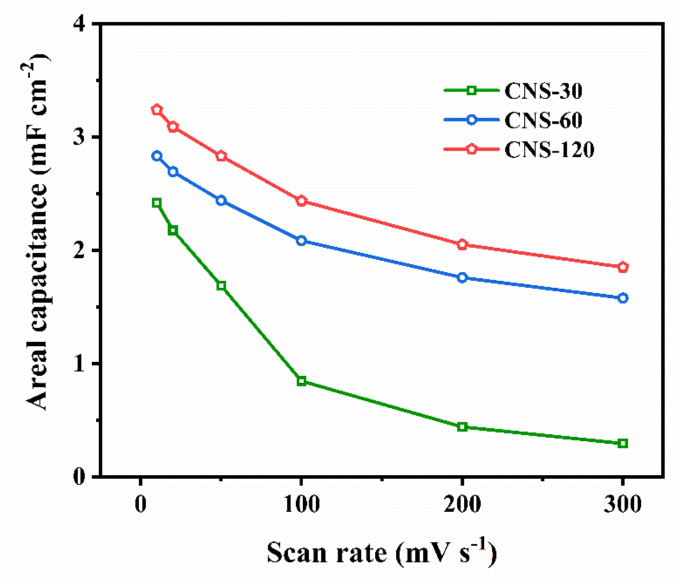

Figure S5. Areal capacitance of CNS-30, CNS-60 and CNS-120 at different scan rates.

Table S2. Comparisons of the electrochemical performance for CNS-120 electrode and previously reported materials electrode for two-electrode system.

Sample	Electrolyte	Cs (mF cm^{-2})	Rate performance (mF cm^{-2})	Cycling stability	Ref.
CNS-120	1 M Et_4NBF_4	6.82 (0.01 mA cm^{-2})	3.07 (2 mA cm^{-2})	96.4 % (5 000, 0.1 mA cm^{-2})	This work
MPC	6 M KOH	6.3 (0.8 mA cm^{-2})	5.1 (4 mA cm^{-2})	91 % (10 000, 2 mA cm^{-2})	[1]
N-doped sucrose carbon	H_2SO_4/PVA	3.9 (5 mV s^{-1})	< 3 (100 mV s^{-1})	96 % (10 000, 15 A cm^{-2})	[2]
3D FC-CNT@P	2 M H_2SO_4/PVA	5.53 (0.1 mA cm^{-2})	4.63 (1 mA cm^{-2})	65.8 % (5 000, 0.8 mA cm^{-2})	[3]
Au-CNT	1 M H_2SO_4/PVA	12 (0.5 mA cm^{-2})	6 (40 mA cm^{-2})	93.6 % (10 000, 10 mA cm^{-2})	[4]
Laser-induced graphene	[PYR14][TFSI]	4 (0.01 mA cm^{-2})	2.55 (0.02 mA cm^{-2})	63 % (3 200, 0.02 mA cm^{-2})	[5]
HCSs	KOH/PVA	6.1 (0.5 mA cm^{-2})	2.1 (2 mA cm^{-2})		[6]
G-CNT-5	H_3PO_4/PVA	9.81 (0.05 mA cm^{-2})	8.05 (0.4 mA cm^{-2})	95.5 % (10 000, 0.1 mA cm^{-2})	[7]
PPy@rGO	H_2SO_4/PVA	15.9 (0.25 mA cm^{-2})	12.4 (2 mA cm^{-2})	79.2 % (5 000, 5 mA cm^{-2})	[8]
Laser-induced graphene	[EMIM][OTf]:PPC =0.5	0.46 (0.03 mA cm^{-2})	< 0.4 (0.4 mA cm^{-2})	98.12% (10 000, 0.1 mA cm^{-2})	[9]

laser-pyrolyzed carbon	H_3PO_4/PVA	0.56 (0.002 mA cm^{-2})	0.3 (0.1 mA cm^{-2})	94.7% (4 000, 0.02 mA cm^{-2})	[10]

Table S3. The ESR and R_{ct} values of all the samples.

Sample	ESR (Ω)	R_{ct} (Ω)
CNS-30	1.04	1553
CNS-60	1.17	15.24
CNS-120	1.16	149.3

References

1. Bhardwaj, A.; Pagaduan, J.N.; Yu, Y.G.; Einck, V.J.; Nuguri, S.; Katsumata, R.; Watkins, J.J. Large-pore ordered mesoporous turbostratic carbon films prepared using rapid thermal annealing for high-performance micro-pseudocapacitors. *ACS Appl. Mater. Interfaces* **2021**, *13*, 61027–61038. https://doi.org/10.1021/acsami.1c16666
2. Bräuniger, Y.; Lochmann, S.; Grothe, J.; Hantusch, M.; Kaskel, S. Piezoelectric inkjet printing of nanoporous carbons for micro-supercapacitor devices. *ACS Appl. Energy Mater.* **2021**, *4*, 1560–1567. https://doi.org/10.1021/acsaem.0c02745
3. Xie, X., Guo, R., Yang, B., Li, H., Yang, F., Shen, B. Stencil-printed electrodes without current collectors and inactive additives on textiles for in-plane microsupercapacitors. *J. Mater. Chem. A* **2021**, *9*, 25042–25050. https://doi.org/10.1039/d1ta07302h
4. Zhou, Y., Cao, C., Cao, Y., Han, Q., Parker, C.B., Glass, J.T. Robust and high-performance electrodes via crumpled au-cnt forests for stretchable supercapacitors. *Matter* **2020**, *2*, 1307–1323. https://doi.org/10.1016/j.matt.2020.02.024
5. Zaccagnini, P., Di Giovanni, D., Gomez, M.G., Passerini, S., Varzi, A., Lamberti, A. Flexible and high temperature supercapacitor based on laser-induced graphene electrodes and ionic liquid electrolyte, a de-rated voltage analysis. *Electrochim. Acta* **2020**, *357*, 136838. https://doi.org/10.1016/j.electacta.2020.136838
6. Yang, X., Li, Y., Zhang, P., Sun, L., Ren, X., Mi, H. Hierarchical hollow carbon spheres: Novel synthesis strategy, pore structure engineering and application for micro-supercapacitor. *Carbon* **2020**, *157*, 70–79. https://doi.org/10.1016/j.carbon.2019.10.008
7. Wang, Y., Zhang, Y., Wang, G., Shi, X., Qiao, Y., Liu, J., Liu, H., Ganesh, A., Li, L. Direct graphene-carbon nanotube composite ink writing all-solid-state flexible microsupercapacitors with high areal energy density. *Adv. Funct. Mater.* **2020**, *30*, 1907284. https://doi.org/10.1002/adfm.201907284
8. Tahir, M., He, L., Yang, W., Hong, X., Haider, W.A., Tang, H., Zhu, Z., Owusu, K.A., Mai, L. Boosting the electrochemical performance and reliability of conducting polymer microelectrode via intermediate graphene for on-chip asymmetric micro-supercapacitor. *J. Energy Chem.* **2020**, *49*, 224–232. https://doi.org/10.1016/j.jechem.2020.02.036
9. Ray, A., Roth, J., Saruhan, B. Laser-induced interdigital structured graphene electrodes based flexible micro-supercapacitor for efficient peak energy storage. *Molecules* **2022**, *27*, 329. https://doi.org/10.3390/molecules27010329
10. Kwon, S., Choi, H.J., Shim, H.C., Yoon, Y., Ahn, J., Lim, H., Kim, G., Choi, K.B., Lee, J. Hierarchically porous, laser-pyrolyzed carbon electrode from black photoresist for on-chip microsupercapacitors. *Nanomaterials* **2021**, *11*, 2828. https://doi.org/10.3390/nano11112828
